# Report of high data rate macromolecular crystallography (HDRMX) meeting, 23 July 2025

**DOI:** 10.1063/4.0001196

**Published:** 2026-06-04

**Authors:** Christine Beavers, Herbert J. Bernstein, Aaron S. Brewster, Max Burian, Nicholas Devenish, Jiaxin Dawn Duan, Daniel Eriksson, Diego Gämperle, Yang Ha, David R. Hall, James Holton, Peter Keller, Loes Kroon-Batenburg, David W. Mittan-Moreau, Yasukazu Nakaye, Daniel W. Paley, Ezra Peisach, Nicholas K. Sauter, Sofia Trampari, Clemens Vonrhein, David G. Waterman, Thomas A. White, Graeme Winter

**Affiliations:** 1 Rigaku Americas, The Woodlands, Texas 77381, USA and Rigaku Corporation, Akishima, Tokyo 196-8666, Japan; 2 Ronin Institute for Independent Scholarship 2.0, c/o NSLS II, BNL, Upton, New York, 11973-5000, USA; 3 Lawrence Berkeley National Laboratory, Berkeley, California, 94720, USA; 4 DECTRIS Ltd., Baden, Aargau, Switzerland; 5 Diamond Light Source, Chilton, United Kingdom; 6 Paul Scherrer Institute, Villigen, Switzerland; 7 Crystallography Group, Australian Synchrotron, ANSTO, Clayton, Melbourne, VIC 3168, Australia; 8 Department of Biochemistry and Biophysics, University of California, San Francisco, California 94158-2330, USA; Division of Molecular Biophysics and Bioengineering, Lawrence Berkeley National Laboratory, Berkeley, California 94720, USA; and Stanford Synchrotron Radiation Lightsource, SLAC National Accelerator Laboratory, Menlo Park, California 94025, USA; 9 Global Phasing Limited, Cambridge, United Kingdom; 10 Universiteit Utrecht, Utrecht, Netherlands; 11 Research Collaboratory for Structural Bioinformatics PDB, Piscataway, New Jersey, 08854-8076, USA; 12 Research Complex at Harwell, UKRI–STFC Rutherford Appleton Laboratory, Harwell, Didcot, Oxfordshire OX11 0FA, United Kingdom; 13 Deutsches Elektronen-Synchrotron DESY, Hamburg, Germany; 14 Northeastern Collaborative Access Team (NE-CAT) Facility, Advanced Photon Source at Argonne National Laboratory, Lemont, Illinois, 60439, USA

## Abstract

This paper is a report of the High Data Rate Macromolecular Crystallography workshop held on 23 July 2025 as part of the 2025 meeting of the American Crystallographic Association in Lombard, IL, USA, 18–23 July 2025. This report summarizes the discussions, questions, action items, and recommendations that arose from the meeting and includes links to the presentations. The sessions were moderated by Aaron S. Brewster and Graeme Winter. There was particularly lively discussion about the possible need for lossy compression as data rates increase, as multimodal experiments become more popular and as research budgets are squeezed.

## I. INTRODUCTION

High Data Rate Macromolecular Crystallography (HDRMX) is a consortium of software developers, detector manufacturers, facility staff, beamline scientists, and users, who meet to discuss and take action relevant to next-generation challenges in data acquisition, transfer, processing, storage, and provenance.

This full-day session consisted of short talks and longer discussion sections, featuring:

• anticipated data rates
• infrastructure for transfer and processing
• plans for long-term storage
• benefits and costs of lossy compression
• metadata standards
• multimodal experiments
• provenance for data, processing, and deposition
• data and metadata standardization

This workshop was part of the 2025 meeting of the American Crystallographic Association in Lombard, IL, USA, 18–23 July 2025. This report summarizes the discussions, questions, action items, and recommendations that arose from the meeting and includes links to the presentations. The sessions were moderated by Aaron S. Brewster and Graeme Winter.

## II. PROVENANCE IN DIFFRACTION DATA, MTZ, AND CIF

Speakers: Ezra Peisach (representing PDB) and David G. Waterman (representing CCP4 and DIALS)^12,13^

Data provenance includes the record of how data were processed and transformed and is a key part of reproducible science. Crystallographic data processing software reads data produced at a light source or other appropriate diffraction instrument, indexes and integrates Bragg spots, creates merged reflection files and associated statistics, and then uses those files to produce atomic coordinates and structure factor files. Note that structure factor files differ from reflection files. Reflection files are raw or processed experimental data derived from the spots in diffraction patterns, directly from the experiment. Structure factors are either experimental or calculated, and they include a phase. If calculated, they are computed from the positions, types, and thermal parameters of atoms in a proposed model using the structure factor equation. A PDB deposition is focused on experimental results and so includes refined atomic coordinates and B factors as well as experimental structure factors or intensities. The PDB has historically had loose coupling between coordinate files and structure factor files, creating inconsistencies. Current challenges include mixed provenance sources, inconsistent metadata, and space group/precision discrepancies. Many depositions are reported as using “unknown” software, partly due to ambiguous pipeline reporting, even though CIFs can accommodate extensive software lists (30+ lines) as long as they are dictionary compliant, unlike the legacy PDB file format limitations.

As the MTZ format is common for intermediate processing during structure solution, work among CCP4, Diamond Light Source, Global Phasing, and PDBe has been ongoing to create an MTZ appendix for provenance. This exploits the fact that MTZ files end with a trailer, so anything beyond that is ignored by existing software. They are creating a lightweight format allowing arbitrary data (e.g., in JSON format) to be appended, which will enable MTZ files to carry complete metadata from data collection through to processing, refinement pipelines, and ultimately public deposition.

The discussion revealed significant technical and logistical challenges around metadata preservation, the economics of connected, comprehensive data systems, and the need for better coordination between software packages and beamline facilities.

**Results and recommendations:**

• The PDB supports a multi-data block structure factor file format where each data producer maintains authoritative control over their data block. Log file “scraping” should be phased out.
• Facilities need to accurately include their metadata, such as beamline and instrument, in the diffraction data headers, so it can be propagated.
• DIALS is developing an automated history tracking system that records timestamps, program names, and versions.

**Action items:**

• Explore options for storing provenance metadata, including MTZ history and new CIF block approaches (e.g. CCP4, Diamond Light Source, Global Phasing and PDBe appendix project).
• Refinement software needs to preserve processing history.
• Move toward archiving scaled + unmerged reflection data (currently only 1878 examples exist, ongoing work in the PDB).

## III. STREAMING

Speakers: Thomas A. White (ASAP::O) and David W. Mittan-Moreau (DIALS)^10,14^

As data rates continue to increase, including in both speed and volume, developers are looking to data streaming from data acquisition systems to computing centers, either local to a beamline or making use of a significant portion of a major facility. Two systems were presented: ASAP::O at DESY and DIALS streaming at ALS.

CrystFEL is being used to process serial crystallography data in real-time at the P11 beamline of PETRA III. ASAP::O is middleware for passing data between pipeline components with a high-performance backend. Key points include:

1. It uses the EIGER stream interface to feed data from the EIGER2 X 16M detector into the ASAP::O system.
2. This is capable of processing 16 MP frames at 133 frames per second (detector's maximum in full frame readout mode) with modest computing resources using a single computer.
3. It includes filtering blank frames, reprocessing, and the “Millepede” algorithm borrowed from high energy physics (used at the Large Hadron Collider) for rapid, precise, and bias-free detector geometry calibration.

For DIALS, work was presented for streaming both XFEL data from LCLS and Eiger data at ALS to be processed on large computing clusters rather than local systems. The prototype was successfully used during a May 2025 beamtime at Advanced Light Source, streaming from the Gemini beamline to a local 120-core system. Future plans include streaming to the National Energy Research Scientific Computing Center (NERSC) at Lawrence Berkeley National Laboratory in California or to the Oak Ridge Leadership Computer Facility (OLCF) at Oak Ridge National Laboratory in Tennessee. The project uses ZeroMQ for message passing in Python with distributed architecture, including load balancing, real-time display, and archiving. There was some concern about long-distance streaming reliability, suggesting raw data should be saved locally at facilities prior to confirmed archiving at remote locations.

**Results and recommendations:**

• In a pure streaming environment, one may need to eliminate reprocessing cycles, potentially storing only processed results rather than raw data for high-throughput applications like pharmaceutical screening, inasmuch as streaming may be antithetical to archiving data for reprocessing. However, typical research scenarios are likely to continue to need to archive diffraction data.
• Standardize on a streaming format for ZeroMQ packets across facilities and consider making DECTRIS's the default. Note that metadata management (run boundaries, sample changes) is critical.

**Action items:**

• Need a two-way converter between CrystFEL and DIALS geometry formats.
• Need streaming emulators for development.
• Investigate the major authentication challenges when streaming user data across facilities, including negotiating firewalls, and the fact that standard encryption methods may create bottlenecks for high-throughput data transfer. Note that ESnet for cloud streaming authenticates at the beginning and end of the streaming processes; it then uses SSL protocols for encrypted transit, avoiding heavy encryption throughout the data stream.^1^

## IV. DETECTOR SPEED

Speakers: Christine Beavers (Rigaku) and Graeme Winter (NE-CAT/Cornell University)^2^

High-speed detectors have multifaceted challenges: while they can produce data rapidly, the key questions are whether systems can keep up with processing and whether users have to wait for results. There is a gap between detector data rates and network capabilities, where data rates consistently lag about 10  years behind network technology growth, due to the time taken to develop detectors. Therefore, we can use accelerating network speeds as much as possible, but, where capacity is outpaced by demand, we can take advantage of the modular nature of fast detectors that feature multi-panel arrangements. Individual module data can be recorded individually rather than forming complete images, especially for high frame rate applications.

Facilities need to recognize the importance of energy-efficient computing and utilize fast processors (such as ARM) and GPUs as tools in real-time analysis. Good fast feedback enables stopping data collection when sufficient quality data are obtained.

**Results and recommendations:**

• Facilities need to invest in high-end networking hardware, not just fast detectors. Detector manufacturers make the mistake of trying to minimize networking costs instead of respecting the need for adequate (at least 20%) network reserve capacity and often recommend readily available technology to reduce risk and complexity, even if it means lagging behind cutting-edge networking speeds. This affects the speed of downstream processing. End-to-end solutions, including networking, computing, archiving, and related infrastructure, need to be fully considered at the beginning of beamline planning and reevaluated as needed. This may require budgeting IT infrastructure as a direct cost instead of as an indirect cost during purchasing.
• Investigate storing module data separately to enable collecting at higher speeds. Use mechanisms such as virtual datasets in HDF5 to represent combined, synchronized images without affecting storage requirements.

## V. COMPRESSION

Speaker: Herbert J. Bernstein^4,5^

Lossy compression is being investigated in the crystallographic community as a way to handle high data rates and long-term archiving in limited computing and storage environments. While the dynamic range of a diffraction pattern can be quite large, most of the image is dominated by smoothly varying background and is essentially empty. Lossless compression preserves 100% of the information using algorithms such as LZW. Lossy compression uses a variety of techniques, including frame summing, which reverses the effect of fine slicing a rotation scan, pixel binning, where adjacent pixels are averaged or summed to produce a smaller image, or wavelet compression, such as JPEG-2000 and Hcompress; the latter of which is originally from the astronomy community. Finally, only the pixels immediately around integrated reflections need to be retained, though this requires very accurate models of the diffraction experiment, including crystal unit cell, orientation, and mosaicity. Overall, in many cases, 300x to 1000x compression ratios can be achieved using lossy compression, resulting in a moderate loss of detail.

Importantly, it is unclear to some participants whether lossy compression is required at their beamlines, given their current and upcoming data needs. The answer is highly dependent on the annual data production of the facility, the data access patterns for real-time and post-experiment processing and reprocessing, and commitments for longevity of data collected. Archiving costs are highly variable and dependent on many factors, especially the access speed, retention period, and reliability required. This is a complex issue in which choices of compression, both lossless and lossy, have always played a role, including recalling that recording crystallographic data as structure factors is a lossy compression. Careful review and consideration are needed in deciding on future directions. Effective lossy compression requires removing noise from data, but this creates problems because current processing software expects to see noise. When noise is removed, software such as background determination may get confused, requiring either adding back artificial noise or modifying processing algorithms. Optimal compression often occurs when the error from lossy compression equals the existing error in the data.

Finally, many users, especially industrial users, contrary to the approach described in Sec. III on streaming, may wish to keep original images for reprocessing, not just the reduced data files, especially for fragment screening experiments. Acceptable compression levels depend in part on the intended use. Single-crystal structure determination might tolerate lossy compression, but diffuse scattering studies have not been researched for compression tolerance. In part, the tolerance for lossy compression depends on available levels of funding for storage and networks.

**Results and recommendations:**

• If lossy compression is used, researchers are advised to be transparent about it. Notably, if lossy data are shared and/or archived, that lossy data probably should have been used for data analysis. If results are derived from non-lossy data, reproducibility may be lost if the non-lossy data are not deposited.
• Consider keeping a small percentage of original images untampered as a failsafe, and consider treating images as “doilies,” i.e., cutting out spots for separate processing while handling the background differently. Determination of the default actions to be taken needs to be carefully considered, or crystallography may find itself in the unfortunate position of high energy physics of having to discard a large portion of its raw data on the basis of decisions made by Field Programmable Gate Arrays (FPGAs).
• Scientists and AIs need to be trained when collecting data to make thoughtful decisions about compression levels, balancing the needs of those who bear storage costs (facilities) and those who benefit from storing all data (scientists).

The discussion repeatedly returned to fundamental questions, which can be considered action items for future discussion:

• Do we need lossy compression at all or will storage solutions such as tape archives or more durable media obviate the need?
• Who decides what compression level to use — facilities or users?
• How long do the data need to be preserved for use by whom?
• Who “owns” the data?
• Should we be collecting data differently rather than compressing existing data?
• How do we resolve the tension between facility and user responsibilities for data management decisions?

On the question of who “owns” the data, John Helliwell has written, “My only comment would be that *Who owns the data?* or *Who controls decisions on the data?* is solvable only by the ESRF method where the PI as applicant for beamtime there has to tick the box “Do you accept the ESRF Data Policy?”.^8^ When I last read this about a year ago, and I heard nothing that has changed it, it is the ESRF who controls what happens to the data namely, automatic release of i.e., access to the raw data of an experiment after 3 years if not published by a PI before. If there is no clarity then it is a facility's “fault” for not having a clear beamtime application wording as per the ESRF's.”

## VI. NEW DETECTORS

Speakers: Yasukazu Nakaye (Rigaku) and Jiaxin Dawn Duan (PSI)^7,11^

Two new detectors were presented as examples of upcoming systems. From Rigaku, the XSPA detector system was discussed, highlighting several key features:

• **Technical Specifications:** The detector offers very fast sustainable frame rates with parallel readout capability, allowing large area detection without losing frame rate or minimum gate time.
• **Performance:** Can achieve 48 nanosecond gate time for single shot images and up to 56 000 frames per second with 2-bit depth (33 000 fps with 4-bit; 8500 fps at 16-bit).
• **Gapless Design:** Uses a redistribution layer technology to eliminate large pixels at chip edges, creating truly gapless sensors that maintain spatial resolution across pixel boundaries.
• **Burst Mode:** Unique feature allowing rapid switching between counters for ultra-fast imaging, with minimum exposure times down to 1.24 *μ*s (nearly one million frames per second).
• **Data Rates**: Generates extremely high data throughput, following the trend of detector data rates increasing with roughly 10-year delays behind network capacity improvements.
• **Applications**: Includes pump-probe modes for simultaneous pumped and unpumped imaging in single experiments.

16M designs exist but are expensive to prototype without committed customers.

Nakaye also mentioned Rigaku's next-generation detector development, focusing on softer x-ray compatibility and vacuum chamber operation, confirmed to work down to 500 eV without electron multiplication (i.e., without the need to generate cascades of secondary electrons).

For PSI, detector implementations across multiple PSI beamlines were discussed, including three beamlines at the synchrotron (one industry-focused, one for room temperature measurements, and one for automated fragment screening) plus one at SwissFEL for femtosecond crystallography:

• **Current Detectors:** Recently upgraded PX3 with Pilatus 4, PX2 with Eiger 2X 16M, and SwissFEL using Jungfrau detectors.
• **Data Processing:** Developed FPGA-based real-time data processing systems to handle massive data streams, including frame indexing and selective data saving, using bit shuffle LZ4 in the HDF5 format with custom FPGA compression for sparse data.
• **Future Preparations:** Presented specifications for the upcoming Matterhorn detector (A6 design submitted, modules expected in about a year) and Jungfrau 2 (still in design phase, estimated 2+ years away).
• **Data Rate Projections:** Current systems generate 2 GB/s per module at 2 kHz; future detectors at full capacity (10 kHz) would produce 10 GB/s per module, equating to 1 PB per day per module if sustained.

The group discussed the practical applications for such high-speed detectors, including fixed-target rotation crystallography with rapid data collection from microcrystals and materials science applications, particularly *in situ* and in operando experiments requiring rapid stimuli-response observations. There was some concern about practical limits, drawing parallels to single-crystal diffractometry, where faster collection does not necessarily improve facility operations, and noting sample consumption rates, including whether sample production can keep pace with ultra-fast data collection.

**Results and recommendations:**

• Facilities should consider use cases for high repetition rate detectors and match detector capabilities to scientific needs.

## VII. ARCHIVING

Speaker: Diego Gämperle (DECTRIS)^9^

Archiving data are a key aspect of FAIR (Findable, Accessible, Interoperable, Reusable) data curation,^15^ and this session discussed data archiving approaches for high-speed x-ray detectors. First, the current DECTRIS data flow architecture was presented, processing 8–16 bit data at up to 160 GB per second on single servers and handling image assembly, corrections (flat field, pixel mask, virtual pixel), metadata addition, and output via stream or HDF5/NeXus files. DECTRIS provides multiple threshold readouts and special 8-bit floating point counters for electron microscopy detectors (3-bit exponent, 5-bit mantissa) and has either implemented or will be implementing lossy and lossless compression modes. Finally, the DECTRIS Cloud Platform has successfully tested uploading 1.2 PB in 72 h (92 GB/s average) and provides multiple data centers across continents with 2.7 TB/s inter-datacenter bandwidth with performance comparable to other leading infrastructures.

Turning to archiving, the discussion noted significant variation in facility approaches, with some facilities using automated systems with fixed storage limits and FIFO deletion and others like LCLS maintaining a 10-year data retention policy. Multiple speakers confirmed they do retrieve archived data for reprocessing, sometimes years later.

**Action items:**

• Standardize approaches to associate data streams with analysis results.
• Better integration of real-time analysis results (like signal pixel detection) into saved data.
• Community coordination on metadata standards for edge computing applications.

## VIII. MULTIMODAL

Speakers: Aaron S. Brewster (LBNL), Max Burian (DECTRIS), and Peter Keller (Global Phasing)^6^

Multimodal data are difficult to represent in machine readable data. Challenges include complex multi-wedge and multi-energy acquisitions, where traditional approaches generate separate master files for each mini-collection, which makes data processing difficult or suboptimal. Software needs to handle interleaved experiments (changing energy every few degrees including possible inverse beam experiments). We need both faithful recording of what happened and an optimal data processing format.

NeXus was presented as a possible solution, a neutron, and x-ray unified standard data format that captures entire experiments including detector, beam, and all instrument components that may be useful for multimodal data. NeXus is FAIR compliant, uses an HDF5 structure with hierarchical organization, and is built from definitions like Lego blocks to describe a variety of applications beyond diffraction. Users can experiment with the format using the DIALS program dxtbx.any2nexus to convert existing data.

For multimodal data, subentries in a NeXus file can be used to handle multiple experiments within one file, allowing synchronization of different data types (e.g., x-ray detector + spectrometer data or multiple wedges as separate subentries). Common parameters associating related experiments could be expressed in a single file. Importantly, we need two views of the same data: a faithful record of how the experiment actually occurred and processing interpretation of how the data should be analyzed. HDF5 hard links can provide both perspectives in one file.

NXem was also shown, a new application definition in NeXus for electron microscopy, repres enting over a year of development effort to handle the complexity of electron microscope instruments. NXem will be part of the next NeXus release.

**Action items:**

• Standardization for multimodal data across beamlines is needed to avoid software having to handle multiple different approaches to the same problem. Note that even basic implementations are lacking; many beamlines still do not properly record fundamental metadata such as beam center positions.

Concrete examples are needed, including:

• Python scripts showing how to write these complex file structures.
• Extending existing reference datasets with multi-wedge examples.
• Focusing on getting basic standard compliance working before tackling complex cases.

## IX. DATABASES

Speaker: Herbert J. Bernstein^3^

This session began with the argument that the core problem about information gathering and dissemination is not about data representation or individual views of information, but rather understanding relationships among data. Dissemination of data are an important issue in modern science.^15^ The main thesis was that relational databases are the only stable, reliable structure capable of handling massive amounts of information, that “there is no alternative”, and that most people's aversion to relational databases leads them toward NoSQL solutions, which is a mistake. Instead, users should put all information into a relational database structure and then work toward using AI to help formulate problems and create necessary substructures to bridge from the relational database to specific problem requirements.

This was accomplished by the late John Westbrook at the Protein Data Bank by creating mmCIF as a direct mapping of a relational database. All PDB information goes into relational tables before being mapped to other formats. For diffraction images, previous work on imgCIF to NeXus mapping was done about 5 years ago, which allows conversion from NeXus data to the CIF structure (a relational format). The important step is to modularize the structure of the relations by normalization. This plays a major role when the data are introduced into archives and a smaller role in dataset-by-dataset data collection but is also helpful there as data rates increase, and parallelism is needed earlier in the workflows. An example is multi-module detectors (Jungfrau 16M), where separate modules write to different files requiring synchronization, which is something MySQL handles automatically.

In the discussion, the potential value of databases for capturing multimodal data were noted, but it was emphasized that the community has had success in creating interchange formats for diffraction experiments. It was also noted that, for pre-planned data collection structures, the linking is often done before or after acquisition, not during, helping to ameliorate the multiple writer problem.

**Results and recommendations:**

• Normalization is the biggest challenge, as it involves breaking large data structures into smaller ones to localize locking. NXmx in NeXus is normalized, meaning there is a mapping from diffraction data to a relational database available. Facilities that are heavily investing in cloud storage (see the American Science Cloud (AmSC) DOE funding call issued in August 2025) should consider storage solutions that allow relational access.

## X. CLOSING DISCUSSION

The final discussion revolved around storage mechanisms, specifically around file systems. Unix computers use POSIX systems, including a structure with files and folders. However, there are limitations in these systems, such as having millions of files in a directory strains listing the contents of the folder. Container files such as HDF5 are a solution but have issues such as the fact that they cannot be read reliably while being written. Note that NFS problems stem from trying to replicate POSIX-like behavior over networks where such guarantees cannot be maintained. Removing POSIX requirements would allow direct exposure of data storage methods without the overhead of making it look like a traditional file system.

This is the main approach behind object stores, such as those used by Amazon S3, which are key-value databases where you can insert, delete, and read objects (but not update them), enabling massive parallelization. This could work with massive diffraction detector data, where data chunks could go directly into object stores with acquisition IDs as bucket names and sequential keys for headers and data frames. The discussion touched on how other scientific communities (electron tomography) are already using “shared access object stores” with metadata in one object and individual images as independent objects, enabling streaming and asynchronous access.

**Results and recommendations:**

• To achieve higher performance, the crystallography community should stop using POSIX file systems for storing primary data. Object stores would be better tools. Object stores do not care about concurrent access patterns; they simply map identities to data blocks using key-value pairs.

HDRMX meets as needed with announcements sent to the CCP4 bulletin board and coordinated through an open Slack channel. Please reach out with any questions or if you would like to join the channel or otherwise get involved.

## Data Availability

Data sharing is not applicable to this article as no new data were created or analyzed in this study.
